# Flexibility of KorA, a plasmid-encoded, global transcription regulator, in the presence and the absence of its operator

**DOI:** 10.1093/nar/gkw191

**Published:** 2016-03-25

**Authors:** Karthik V. Rajasekar, Andrew L. Lovering, Felician Dancea, David J. Scott, Sarah A. Harris, Lewis E.H. Bingle, Manfred Roessle, Christopher M. Thomas, Eva I. Hyde, Scott A. White

**Affiliations:** 1 School of Biosciences, The University of Birmingham, Edgbaston, Birmingham B15 2TT, UK; 2 School of Cancer Studies, The University of Birmingham, Edgbaston, Birmingham B15 2TT, UK; 3 School of Biosciences, Sutton Bonington Campus, University of Nottingham, Nottingham LE12 5RD, UK; 4 School of Physics and Astronomy and Astbury Centre for Structural and Molecular Biology, University of Leeds, Leeds LS2 9JT, UK; 5 EMBL, DESY, 22607 Hamburg, Germany

## Abstract

The IncP (Incompatibility group P) plasmids are important carriers in the spread of antibiotic resistance across Gram-negative bacteria. Gene expression in the IncP-1 plasmids is stringently controlled by a network of four global repressors, KorA, KorB, TrbA and KorC interacting cooperatively. Intriguingly, KorA and KorB can act as co-repressors at varying distances between their operators, even when they are moved to be on opposite sides of the DNA. KorA is a homodimer with the 101-amino acid subunits, folding into an N-terminal DNA-binding domain and a C-terminal dimerization domain. In this study, we have determined the structures of the free KorA repressor and two complexes each bound to a 20-bp palindromic DNA duplex containing its consensus operator sequence. Using a combination of X-ray crystallography, nuclear magnetic resonance spectroscopy, SAXS and molecular dynamics calculations, we show that the linker between the two domains is very flexible and the protein remains highly mobile in the presence of DNA. This flexibility allows the DNA-binding domains of the dimer to straddle the operator DNA on binding and is likely to be important in cooperative binding to KorB. Unexpectedly, the C-terminal domain of KorA is structurally similar to the dimerization domain of the tumour suppressor p53.

## INTRODUCTION

The IncP (Incompatibility group P) plasmids are important carriers of antibiotic resistance. They are low-copy-number plasmids that can transfer to and be stably maintained in almost all Gram-negative bacteria, as well as being able to transfer to some Gram-positive bacteria and higher eukaryotic cells. Gene expression of all the plasmid backbone functions, such as replication, partitioning and plasmid transfer, is stringently controlled by a network of four plasmid-encoded repressor proteins: KorA, KorB, KorC and TrbA (1). Two repressor proteins bind to most core promoters. KorB also plays a role in plasmid partitioning as the ParB (DNA-binding protein) homologue. The KorA protein from the IncP1 plasmid RK2 binds with KorB at five of its seven operator (O_A_) sites (2) and they have been shown to act cooperatively at two of these (3). This cooperativity involves the C-terminal domain (CTD) of KorA, which has a 77.4% sequence similarity to that of TrbA (Supplementary Figure S1), that also acts cooperatively with KorB (4). Key operons controlled by KorA and KorB acting together include the operons involved in vegetative replication and conjugative gene transfer, and the central control region, encoding KorA, KorB and IncC (the ParA homologue) required for plasmid partitioning. KorA appears to be uniquely responsible for controlling gene switching between the two different modes of plasmid DNA replication (5,6). Intriguingly, KorB is able to repress and act cooperatively at distances more than 1000 bp upstream or downstream from the binding site of the repressor TrbA (7), similar to the effects of eukaryotic enhancers. Cooperative binding also occurs between KorB and either KorA or TrbA at short distances, and surprisingly the interaction remains cooperative even when 5 bp, introducing a twist of 180°, is inserted between the two binding sites (7). The effects at very short distances cannot be explained by DNA looping while the long-range effects cannot be explained by the protein spreading along the DNA.

In order to understand the molecular basis of the cooperativity between KorA and KorB, we are examining their structures and interactions. KorA is a 101-amino acid, homodimeric, protein with an N-terminal DNA-binding domain (DBD), containing a helix-turn-helix motif, and a C-terminal dimerization region (3), joined by a linker of four amino acids. A crystal structure of KorA bound to a 18-bp operator (O_A_) DNA has been determined previously (8). Here we examine the structure and mobility of KorA in the absence of DNA and structural changes upon DNA binding, using a combination of crystallography, nuclear magnetic resonance (NMR) spectroscopy, small angle X-ray scattering (SAXS) and molecular dynamics (MD). We show that the DBDs significantly change orientation upon DNA binding and that the CTD is mobile, due to the highly flexible linker, both in the free protein and DNA-bound complexes. This flexibility is likely to contribute to the cooperativity between KorA and KorB at different distances. Surprisingly, despite the lack of sequence homology, the CTD is structurally similar to the dimerization domain of the tumour suppressor p53.

## MATERIALS AND METHODS

### Protein expression and purification

The *korA* gene from RK2 was expressed and the protein purified as described previously (9). ^13^C- and/ or ^15^N- labelled protein was expressed and purified from cells grown in M9 minimal medium with ^15^NH_4_Cl and ^13^C_6_H_12_O_6_ as the sole nitrogen and carbon sources, respectively.

### DNA

A palindromic oligonucleotide, containing the sequence:

C_1_. C_2_. A_3_. A_4_. G_5_. T_6_. T_7_. T_8_. A_9_. G_10_. C_11_.T_12_.A_13_.A_14_.A_15_.C_16_.T_17_.T_18_.G_19_.G_20_ was purchased from MWG-Biotech AG. This is the consensus 12 bp O_A_ sequence (underlined), plus two additional flanking base pairs from the strongest O_A_ site, plus two CG base pairs at either end. It was annealed in buffer containing 10 mM Tris–HCl pH 7.0, 1 mM ethylenediaminetetraacetic acid (EDTA), 100 mM NaCl.

The KorA–O_A_ complex (∼1 mM) was made by mixing KorA dimers and the operator at a ratio of 1:1.05 and dialysed into 20 mM Tris pH 7.0, 100 mM NaCl, 1 mM EDTA. For NMR studies the complex was made in 20 mM sodium phosphate buffer pH 6.2, 100 mM NaCl and 1 mM EDTA.

### X-ray crystallography

Crystals of unbound KorA were grown by vapour diffusion with 20 mg.ml^-1^ protein mixed 1:1 with reservoir consisting of 200 mM sodium acetate, pH 4.5, 200 mM ammonium acetate and 32.5% w/v PEG-4K. KorA–O_A_ crystals were grown by vapour-diffusion with 14 mg.ml^-1^ KorA–O_A_ complex mixed 1:1 with reservoir consisting of 4–8% PEG-6K, 100 mM sodium acetate, pH 4.6 and 25% ethylene glycol. Crystallographic data (Supplementary Table S1) were analysed with XDS (10), CCP4 (11) and PHENIX programs (12). The structure of free KorA was phased using MIRAS with phase improvement using SOLVE/RESOLVE (13). Two structures of DNA-bound KorA–O_A_ complexes were determined by molecular replacement (PHASER (14)) using the DBD and CTD from the free structure as separate search models, together with a theoretical model of O_A_. All models were built and improved using COOT (15), and structures were refined using both REFMAC (16) and PHENIX.REFINE (12).

### NMR data acquisition and analysis

NMR relaxation experiments were recorded on a Varian Unityplus 600 MHz spectrometer. NOESY-^15^N-HSQC and NOESY-^13^C-HSQC were recorded on Varian 800 MHz NMR spectrometers. All experiments were done in 20 mM sodium phosphate buffer pH 6.2, 100 mM NaCl and 1 mM EDTA at 298K or 303K for the free protein and at 308K for the protein–DNA complex. Spectra were processed with NMRPIPE software (17) and analysed using CCPNMR (18). ^15^N and ^1^H chemical shift differences between free protein and DNA-bound amide resonances of KorA, combined using the formula δ comb^2^ = (0.15 δ ^15^N)^2^ + (δ ^1^H)^2^ (19).

Amide-^15^N relaxation data were collected using the method of Kay *et al*. (20). Peak heights for T_1_ and T_2_ data were fitted to a single exponential, F = a.e^−bx^, using CCPNMR software (18). The programme TENSOR2 was used to determine the correlation times for the two domains separately, assuming isotropic tumbling (21).

### Structure determination by NMR spectroscopy

The backbone dihedral angles were predicted from the chemical shifts using TALOS (22). Initially 1200 NOEs were assigned manually from the ^15^N NOESY–HSQC spectrum and 460 NOEs assigned in the ^13^C NOESY-HSQC. Automated assignment of the remaining NOEs was performed using the symmetric dimer protocols of ARIA (23), with an initial torsion angle dynamics (TAD) step followed by MD—simulated annealing (SA) procedure, starting from the protein structure in KorA–O_A_ Complex 1 (PDB ID: 5CM3). In addition, four hydrogen bonds at the dimer interface, supported by the NOE patterns, were imposed throughout the iterative assignment stages. This led to a final list of 2588 unambiguous and 124 ambiguous NOE-derived distance restraints, including duplicates.

The final structure calculations were carried out from a random coil structure using the assigned NOE and dihedral angles, with the same TAD MD-SA protocol, along with two additional Cartesian dynamics cooling phases. The 20 energetically best structures from the calculated 200 structures were refined in water (24), during which no symmetry restraints were imposed (Supplementary Table S2). The median NMR model was calculated by a maximum likelihood superpositioning using the programme THESEUS (25).

### Small angle X-ray scattering data acquisition and analysis

SAXS data were collected at Beamline X33 at DESY (Hamburg), using three sample concentrations 2.5 mg.ml^−1^; 5 mg.ml^−1^ and 10 mg.ml^−1^, in 20 mM Tris–HCl pH 7.0, 100 mM NaCl 1 mM EDTA buffer, at 293K. The datasets from the three samples were merged using the programme PRIMUS (26). The data were confirmed by SEC-SAXS, at Beamline BM29 at ESRF (Grenoble). About 50 μl KorA at 4 mg.ml^-1^ was loaded onto a Superdex 200 Increase (3.2/300) column equilibrated with the above buffer, coupled to the SAXS flow cell. The Guinier plot was used to determine the radius of gyration, R_g_ and the intensity at zero angle, I_0_, which was compared to that of bovine serum albumin to give the molecular mass of the protein. The scattering data were then examined using the dimensionless Kratky plot (27) of (qR_g_)^2^I_q_/I_0_ versus qR_g_ where q is the scattering vector. The distribution of conformers was determined using the EOM 2.0, Ensemble Optimization Method (28,29), to fit the scattering data, using the X-ray structures of the CTD dimer and two DBDs with the 4 aa residue linkers remaining flexible to generate the ensemble. In addition, the pair distance distribution function (P(r) versus r) was generated using the programme GNOM (30), followed by shape reconstruction using 10 rounds of the programme GASBOR (31) to give bead models of the protein. Final reconstructed shapes were compared and averaged using the programme DAMAVER (32) and presented as an envelope using SITUS (33). In parallel, the programme BUNCH (34) was used to find the orientation of the protein domains that fitted best to the GNOM output, based on the X-ray structures of the CTD dimer and the two DBDs with 4 aa residue flexible linkers between the domains.

### Molecular dynamics calculations

All MD calculations used the AMBER 10 suite of programs (35). Simulations of the KorA–O_A_ complex were performed using Complex 1 (PDB ID: 5CM3) as starting coordinates; containing residues 1–97 for one monomer and 1–93 of the second. The DNA was removed from this structure to perform simulations of the KorA dimer alone. The protein and protein/DNA complex were neutralized using Na^+^ counterions, before sufficient TIP3P water molecules were added to ensure 10 Å of water in each orthogonal direction around the solute. The PARM99SB force field (36) was used to describe the protein, and the PARM99 force field (37), in conjunction with the PARMBSC0 forcefield modification (38), was used to describe the DNA. The system was energy minimized and then equilibrated using a standard multi-stage protocol. The temperature was maintained at 300 K using the Berendsen weak coupling scheme (with a coupling constant of 1 ps) and constant pressure (1 atm) using volume scaling (with a coupling constant of 0.2 ps). Long-range electrostatics interactions were treated using the Particle Mesh Ewald technique. The SHAKE algorithm was used to constrain bonds to hydrogen, allowing a 2 ps time step to be used to integrate the equations of motion. MD simulations were performed for 100 ns. Conformations were recorded every 1 ps for further analysis. The trajectories were visualized using VMD and CHIMERA (39,40).

## RESULTS

### Structure of free KorA

**Figure 1.**
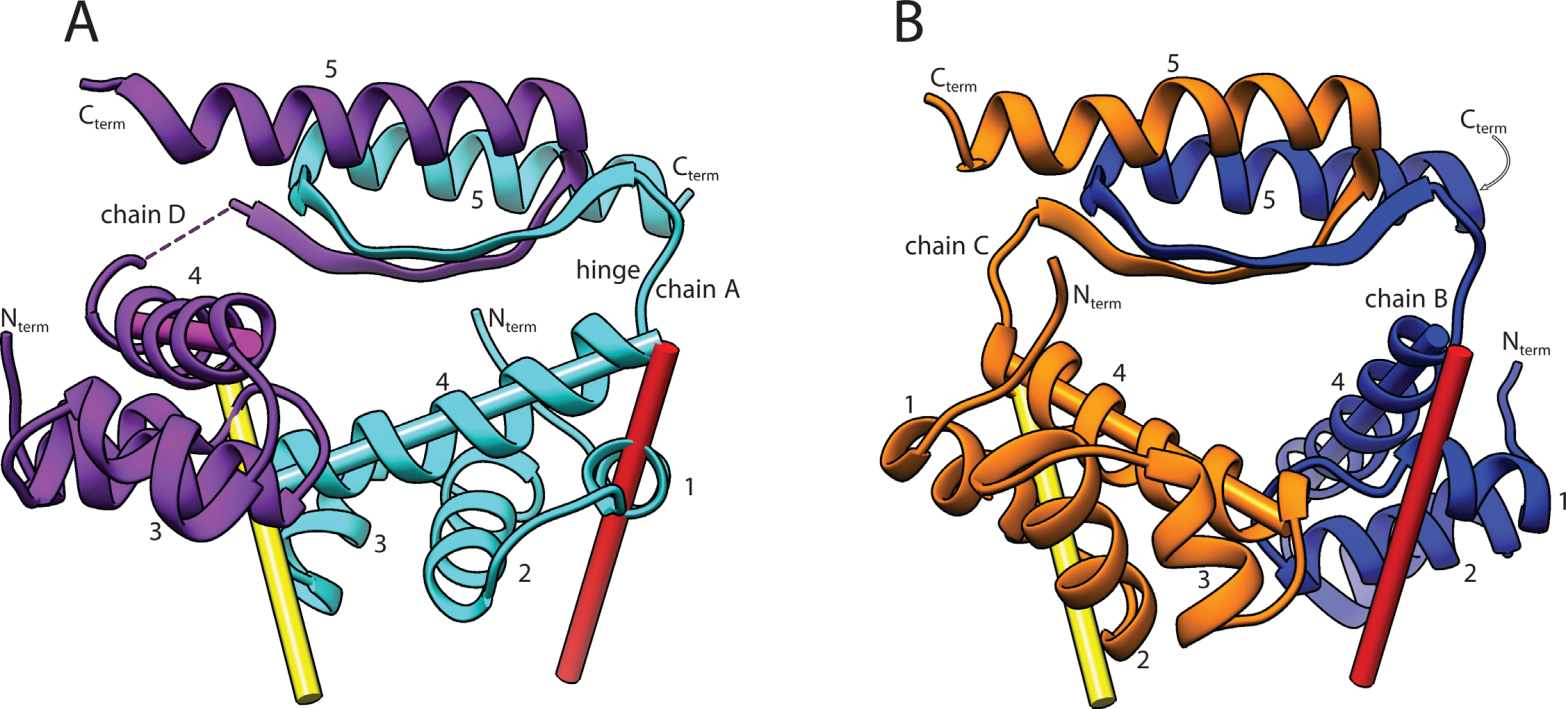
Ribbon diagrams of the X-ray structure of the free KorA dimers (PDB ID: 5CKT). Each subunit is labelled chain A–D at the corresponding flexible hinge region and at the N- and C-termini, while the helices are numbered 1–5 in the N to C direction. The recognition helices of the DNA-binding helix-turn-helix motifs are highlighted with a cylinder of the appropriate colour. The red and yellow cylinders indicate the approximate position of the corresponding helices in the KorA–DNA complex (PDB ID: 5CM3). (**A**) KorA ribbon diagram for Subunits A (cyan) and D (purple). The hinge of chain A is labelled. There is no electron density for the hinge region (residues 65–69 inclusive) in Subunit D, indicating disorder. (**B**) KorA ribbon diagram for Subunits B (blue) and C (orange).

The crystals of free KorA contained two dimers in the asymmetric unit (ASU), each with two N-terminal DNA Binding Domains with four α-helices and a C-Terminal Dimerization domain containing a single β-strand and the final helix from each monomer (Figure 1). The secondary structure of each dimer is similar to the DNA-bound forms (PDB ID: 2W7N (8) and 5CM3, 5CLV, this work), but the orientation of all four copies of the DBD relative to the CTD differs greatly, both from one another and from their positions in the DNA-bound complexes. This is shown by the solid cylinders in Figure 1 which represent the positions of helix 4, the DNA-recognition helix, in the free proteins and in the protein–DNA complexes shown in Figure 3. This variability in orientation is due to differences in conformation of the four amino acid linker between the domains (residues 66–69). This linker is highly flexible, so much so that the electron density of the linker in subunit D is not seen in the crystal structure.

**Figure 2.**
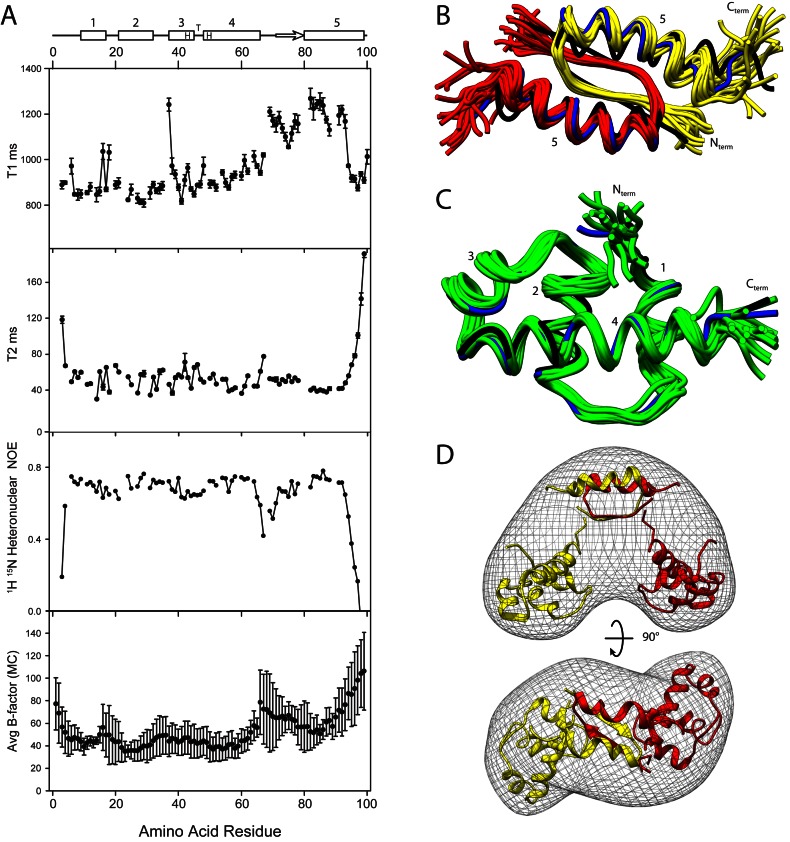
NMR data for free KorA. (**A**) Secondary structure assignment with the 5 α-helices shown as rectangles, marked 1–5 and the β-strand shown as an arrow (HTH indicates the position of the helix-turn-helix motif); ^15^N T_1_ and T_2_ relaxation times; ^1^H-^15^N heteronuclear NOEs; and main chain B factors averaged over each residue of KorA in chains A–D (PDB ID: 5CKT). The error bars for the T1 and T2 measurements are the error in fitting the intensities to a single exponential, while for the B factors they show the variation in B factors for the four chains. (**B**) Superposition of 20 lowest energy NMR (backbone) structures for the CTD of KorA, Subunit A (red) and Subunit B (yellow). Helix 5 is numbered. The median NMR model as calculated by THESEUS is shown in blue. Also shown is the CTD of the free KorA structure (PDB ID: 5CKT) in black. (**C**) Superposition of 20 lowest energy NMR structures for the N-terminal DBD of KorA (green). The median NMR model is shown in blue. The DBD of 5CKT:A is shown in black. The termini and the four helices are indicated. (**D**) Two orientations of the GASBOR bead model of electron density that fits the SAXS distance distribution plot. For clarity the bead model is shown as an envelope with the programme SITUS (33). Superposed is the ribbon model of the optimum orientation of the X-ray structure of the domains fitted to the same distribution using the programme BUNCH (34).

Because of the variable orientation of the domains, NMR spectroscopy was used to examine the structure and dynamics of the protein in solution. The NMR spectrum of KorA was assigned previously (41), only one signal is seen for each NH group in the ^15^N-^1^H HSQC spectrum confirming that the protein is symmetrical in solution on the NMR time scale (Supplementary Figure S2). ^15^N T_1_, and T_2_ relaxation times, and heteronuclear ^15^N-^1^H NOEs of the main chain amide groups were measured (Figure 2A). The T_1_ relaxation times for residues 69–93 are longer than for the N-terminal residues (apart from residue Q37 which is key for DNA binding) while the T_2_ values show the major variation at the ends of the molecule. The T_1_/T_2_ ratios suggest that the DBD of KorA has a shorter rotational correlation time (t_c_ 11.9 ± 0.2 ns) than the CTD (t_c_ 13.5 ± 0.31 ns), indicating that the DBD has some additional independent motion. The ^1^H-^15^N heteronuclear NOEs are similar throughout the molecule except at the termini and internally around the linker at residues 66–71. The lower NOEs show that these N-H groups undergo faster internal motion than the others. The NOEs correlate with the X-ray crystallographic B factors for the protein and confirm that the linker is flexible.

**Figure 3.**
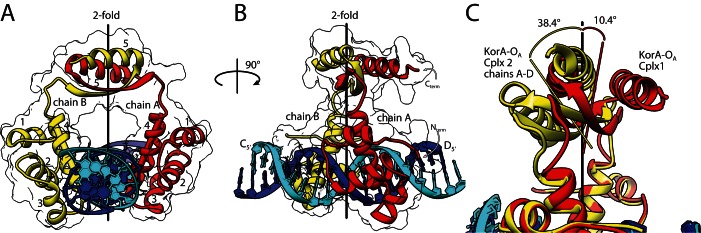
X-ray crystal structures of KorA bound to a 20-bp oligonucleotide containing the O_A_ consensus sequence. (**A**) KorA–O_A_ Complex 1 (PDB ID: 5CM3)- ribbon diagram with silhouette of protein surface and local 2-fold axis calculated using the DBDs of Subunits A and B. The protein is coloured: Subunit A in red, B in yellow. Chains C and D of the operator DNA are coloured cyan and blue, respectively. Helices are numbered 1–5 for each chain. The recognition helices of subunits A and B are highlighted using solid cylinders (see also Figure 1). (**B**) PDB ID: 5CM3, rotated 90° from A about the local 2-fold axis. Chains C and D are labelled at their 5′ ends. (**C**) Part of chains A–D of KorA–O_A_ Complex 2 (PDB ID: 5CLV) depicted in yellow (protein ribbon) and cyan (DNA)) superimposed onto chains A and B of Complex 1 (PDB ID: 5CM3) shown in red (protein ribbon) and blue (DNA)). Also shown are local 2-fold axes calculated from the CTDs of 5CLV (yellow) and 5CM3 (red) and the angular displacement from the DBD 2-fold axis.

The structure of each domain is well-determined from the NMR data (Figure 2B and C), and is consistent with the crystal structures (Figures 1 and 3). The structures of the DBD (residue 6–66) from both techniques fit with an RMSD of 0.92 Å, while the CTD structures (residues 71–98, dimer) fit with an RMSD of 2.9 Å. However, the relative orientation of the domains could not be determined by NMR as no NOEs were observed between the two domains, nor between the resonances of residues in the loop and the domains, indicating a high degree of disorder. Moreover, the chemical shifts of the residues 65–70 did not give strong backbone angle constraints, suggesting averaging of their conformation. We therefore turned to SAXS (Supplementary Figure S3) to examine the conformational range of the protein in solution. The scattering intensity gave a linear Guinier plot, showing that the protein was monodisperse, with the scattering intensity at zero angle giving a molecular mass of 21.3 kDa, within error of the calculated value for a dimer of 22.6 kDa. The dimensionless Kratky plot (27) of (qR_g_)^2^ I_q_/I_0_ versus qR_g_, shows a peak with a maximum at coordinates (*√3, 3/e*), as expected from a folded protein. However, there is also a second maximum, (approximate coordinates (4.8, 0.83)) and the plot did not go to zero, indicating that the protein has two folded domains and a flexible linker (Supplementary Figure S3D). Analysis of the scattering curve using EOM (ensemble optimization method) (28,29), shows that the protein adopts mainly compact conformations (Supplementary Figure S3E). As such, it can be useful to find a single conformation that represents an average position of the domains (42). Figure 2D shows a surface representation of a bead model of the protein based on the SAXS data, assuming P2 symmetry, using the programs GASBOR (31) and SITUS (33) while the ribbon models show the fitting of the separate domains to the SAXS data using BUNCH (34). In different BUNCH calculations, the domains rotate within the distribution but the average distance between the domains is maintained, giving a clover leaf structure.

### Structure of KorA bound to O_A_ DNA

KorA was crystallized in the presence of the operator O_A_. In contrast to the free protein, several crystal forms of the complex were obtained. Structures from two of these were determined. Both show the DBDs of the protein symmetrically placed about the DNA axis (Figure 3). In contrast, the orientation of the CTD differs in the two crystal structures. In the first complex (PDB ID: 5CM3), containing a single DNA-bound dimer in the ASU, the CTD is rotated only 10.4° from the local 2-fold axis. In Complex 2 (PDB ID: 5CLV), there were four copies of the dimeric protein–DNA complex in the ASU. However, only one CTD could be modelled, although all eight DBDs are clearly visible. There is only weak, partial electron density for the other three CTDs, presumably as they are highly disordered. In addition, the one CTD that is observed in 5CLV is tilted 38.4° away from the local 2-fold axis (Figure 3).

The protein–DNA contacts in each of these two structures are similar to each other (Supplementary Figure S4) and to that of PDB ID: 2W7N, determined previously, (8). In contrast to most proteins containing a helix-turn-helix motif, that bind to one face of the DNA; the two DBDs of KorA straddle the DNA, with helix 4 of the two subunits binding to opposite faces of the major groove (Figure 3 A and B). As in PDB ID: 2W7N (8), Q53, in the centre of helix 4, appears to be the key residue for DNA recognition as it contacts three bases, two adjacent Thymine bases on opposite strands of the DNA (T8 and T12) via its side chain amide group, and a third base (Cytosine 11) via the sidechain carbonyl group. In addition R48, at the beginning of helix 4, binds to Guanine 5 O6, but an alternative conformation of its sidechain is also seen in one half site, binding to phosphate 4. G49 is positioned so that its Cα atom is in van der Waal's contact with Thymine 7-O4. This restricts both the amino acid identity and the base, as other bases, including Cytosine, would cause a steric clash. The hydroxyl group of T47 forms a water-mediated contact with Adenine 13 in both chains. The remaining protein–DNA contacts are to the phosphate groups, including many from residues 18–23, outside the helix-turn-helix motif. There are small differences in the contacts between the subunits, eg. R57 in helix 4 contacts phosphate 9 in one chain and phosphate 10 in the other.

**Figure 4.**
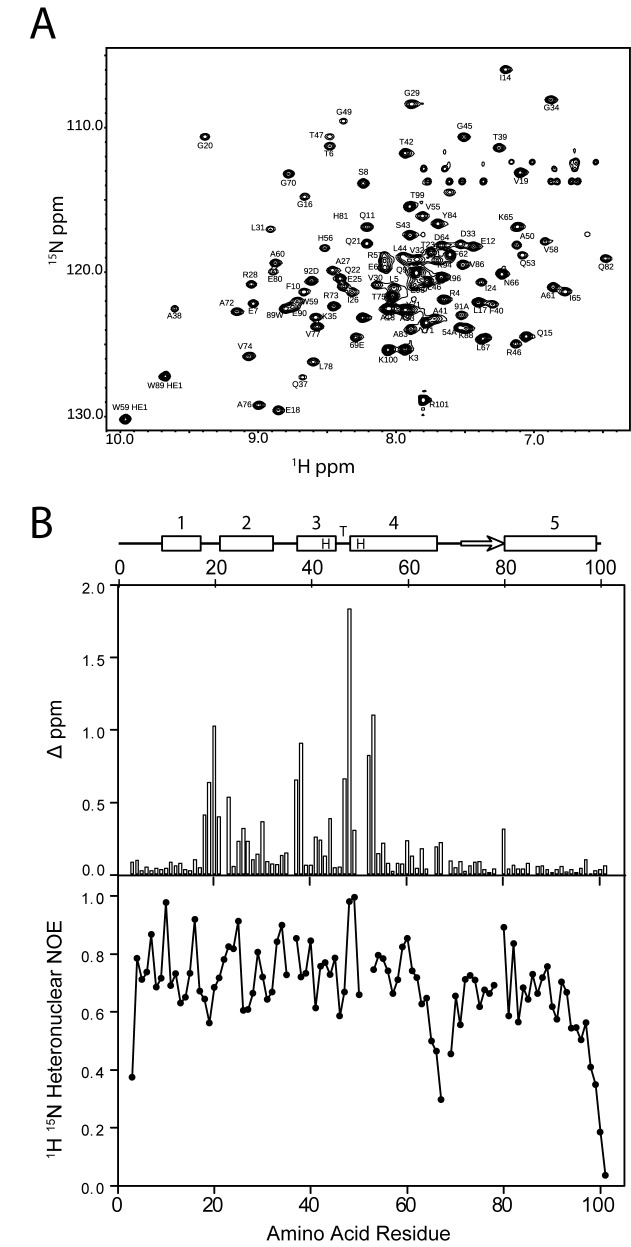
NMR data of KorA bound to a 20-bp oligonucleotide containing the O_A_ consensus sequence. (**A**) ^15^N-^1^H HSQC of KorA–O_A_ at 308K recorded at 600 MHz. The peaks are labelled with the amino acid assignments of their NH group, the two peaks at ∼10 ppm are from the indole NH groups of the two tryptophan residues. (**B**) Top: schematic secondary structure of KorA, the boxes show the α-helices, which are numbered and the arrow shows the β-strand. HTH indicates the position of the helix-turn-helix motif. Centre: combined ^15^N and ^1^H chemical shift differences between free protein and DNA-bound amide resonances of KorA. Bottom: ratio of peak intensities of ^15^N-^1^H HSQC spectra of the KorA–O_A_ complex taken without and with proton saturation of 2.9 s, giving heteronuclear ^15^N-^1^H NOE.

Because of the differences seen in the crystal structures, we examined the mobility of the protein–DNA complex in solution, by NMR spectroscopy. In the NMR spectra the signals of bound and free protein are in slow exchange, showing tight binding. The ^15^N-^1^H HSQC spectrum of the complex (Figure 4A) was assigned using sequential NH-NH and NH-CαH NOEs. The differences in chemical shifts between the ^15^N and ^1^H amide resonances in the free and DNA-bound protein are shown in Figure 4B. The CTD and the first 17 amino acid residues show minimal changes in chemical shift on binding DNA, suggesting that they are unaffected by DNA binding. However changes in shifts are seen for the remaining residues, with the largest effects at residues 18–23, 37–38 and 47–53, in agreement with these NH groups being close to the DNA, as in the crystal structures. Heteronuclear ^15^N-^1^H NOE measurements of the KorA–O_A_ complex again show a dip in the NOE intensity for residues in the linker region, showing that the linker retains some flexibility in the presence of DNA (Figure 4B).

**Figure 5.**
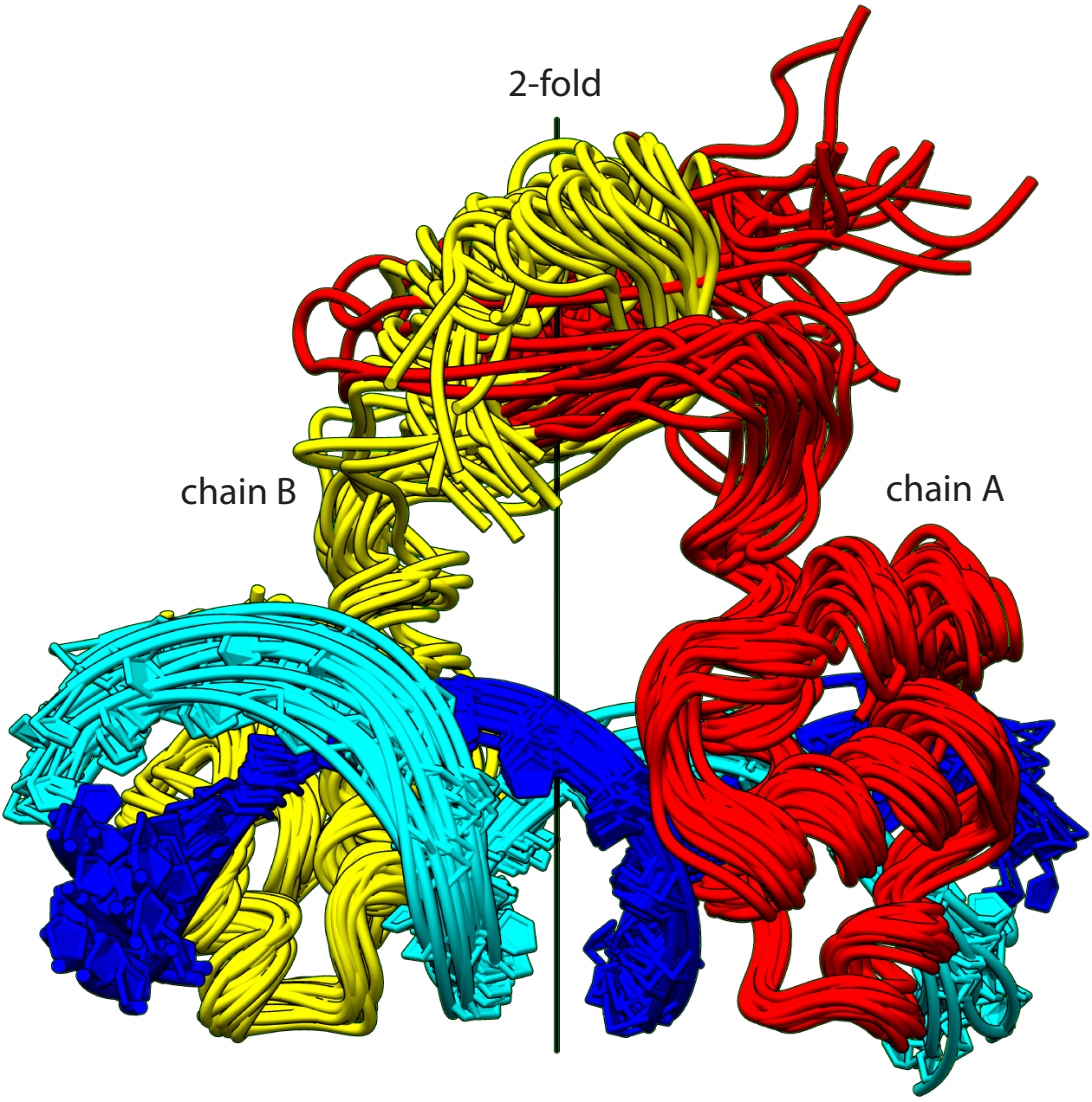
Molecular dynamics (MD) data. Overlay of 20 structures of the KorA–O_A_ complex, sampled over 100 ns of the MD calculation. The protein backbone is coloured red and yellow for subunits A and B; with the DNA strands in blue and cyan.

Given the highly flexible structure of the protein, MD simulations were used to explore the conformations of the free and bound protein in solution. Figure 5 shows an overlay of the conformers sampled for the KorA–DNA complex, over 100 ns of simulation; the complex remains very flexible with the CTD exploring a wider range of orientations than the two observed in the crystal structures (Supplementary Movie 1). When the protein is removed from the DNA complex, the DBDs collapse towards each other, giving additional fluctuating contacts which keep the protein compact (Supplementary Movie 2). Dynamic interactions observed between the two DBDs include salt bridges between R57 and E18 and hydrogen bonds between R48 and Q11/Q15. In the DNA complex these interactions are replaced: R57 and E18 both bind to the phosphate backbone, while R48 binds to Guanine 5. The relatively high T1 values for G16 and E18 in the NMR studies of the free protein may reflect their dynamics.

## DISCUSSION

The structure of KorA consists of two domains, joined by a highly flexible, four amino acid linker, which retains its mobility even when bound to DNA. Only by combining a suite of complementary biophysical techniques (X-ray crystallography, NMR spectroscopy, SAXS and MD) was it possible to study this system due to its inherently dynamic nature. The mobility of the KorA DBDs, in the absence of DNA, is likely to be due in part to the different possible electrostatic interactions between the two domains, giving rise to multiple conformers of similar energy. In addition, the recognition helix of KorA, helix 4, is longer than in classical helix-turn-helix proteins and so the CTD is held away from the operator DNA and it has no interactions with the DBDs, making it flexible even in the presence of DNA.

The consensus operator sequence of the protein covers 12 bp (G5-C16 in our sequence) and is found in the three tightest binding KorA operators (2). Three of the remaining four operators do not have Guanine at position G5 while the last has C8 instead of T8, and these four operators all bind more weakly. In our structures, as in PDB ID: 2W7N, determined previously (8), the base pairs outside the consensus sequence do not form any base contacts to the protein, despite the preference of AT base pairs here in the natural operators giving increased binding *in vitro*. In each operator half site, five of the six consensus base pairs form direct hydrogen bonds to a protein side chain. The bases of T6-A15 form no direct bonds to the protein but the phosphates 6 and 7 form a number of direct and water-mediated interactions, which likely restrict the conformation. Our structure shows essentially the same contacts as in Konig *et al*; however, in 2W7N the DNA is fitted in two orientations each with 0.5 occupancy, giving averaged protein-DNA contacts while in 5CM3 and 5CLV minor differences are observed in the DNA–protein contacts between the two half-sites within the palindromic operator, such as for the side chains of R48 and R57, again suggesting a dynamic interface.

**Figure 6.**
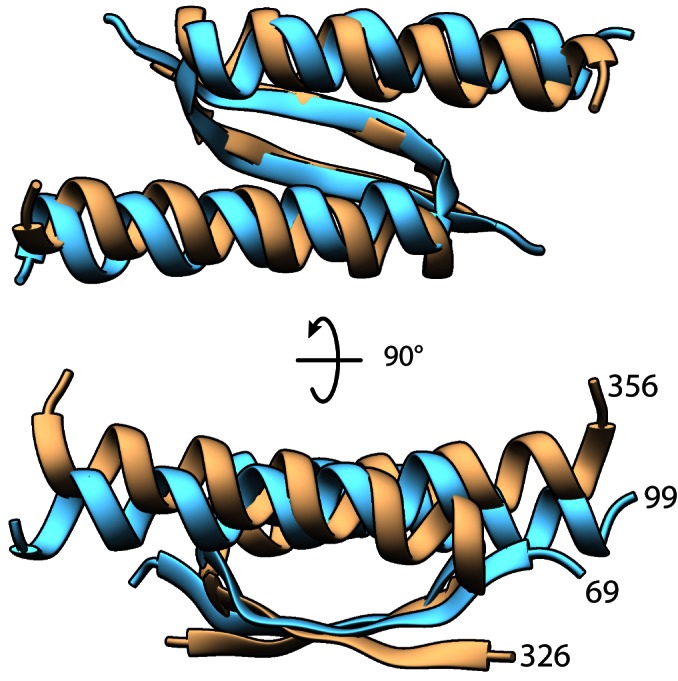
Comparison of CTDs of KorA (blue) and dimerization/tetramerization domain of p53 (tan, PDB ID: 1AIE (43)).

While examining the structure of the protein, we observed a structural similarity between the CTD of KorA and that of p53 (PDB ID: 1AIE (43), Figure 6), that was not noted previously. There is no sequence similarity between the two proteins. However, in both proteins this 31-residue domain consists of a nine-residue β-strand, a three-residue turn and a 19-residue α-helix. Like p53, which has long flexible linkers, and can be considered as a dimer of dimers (44), the KorA dimer binds across the DNA duplex rather than to one face of the DNA. The mobility between DBD pairs may be necessary to allow KorA and p53 to bind in this mode. Most proteins that straddle DNA are either unstructured in the absence of DNA (such as the basic region of GCN4 (45)) or only form dimers when bound to the DNA (such as Fos and Jun (46)).

The KorA and p53 CTD surfaces differ in electrostatic properties and shape (Supplementary Figure S1). While p53 CTD forms homotetramers through a four-helix bundle, KorA CTD forms dimers. However, the CTD of KorA is involved in interactions with the co-regulator KorB, as shown by mutational analysis and NMR spectroscopy (9). The mobility of KorA CTD while bound to DNA allows it to flex considerably towards KorB. Intriguingly, KorB, is also a highly flexible protein, with two regions of intrinsic disorder, as well as two structured domains (47). The flexibility within KorA and KorB, even when bound to DNA, allows them to contact each other at different distances and relative orientations (7). Similar cooperative interactions at variable distances are a feature of many eukaryotic transcription factors which also often contain intrinsically disordered regions (reviewed in (48)), such as the transcription activator domains of yeast Gal4 and HMGA family proteins such as SOX15 that bind to the minor groove of AT-rich DNA; as well as p53, and Fos and Jun, mentioned above.

The sequence of the linker (residues 66–69) in KorA is NLPE. Sequences of 92 KorA proteins, with sequence identities >37% were aligned using BLAST (49). The alignment of residues 45–79, covering the DNA recognition helix, the linker and the beta strand shows high sequence conservation of amino acids at the beginning of the DNA recognition helix of the protein to residue 59 and a preference for A at position 61 (Supplementary Figure S5). There is also high sequence conservation from residue 70–89 in the C-terminal domain. There is much less sequence conservation of residues 62–66, that do not contact the DNA but make this helix longer than in a classical helix-turn-helix motif (50), while in the linker, P68 is conserved across more than 97% of KorA sequences, and there is a preference for V or L at position 67. This suggests that the length of the recognition helix, but not the sequence of the last turn, together with a short linker centred at P68, may be important for KorA function.

Intrinsically disordered regions of 20 or more amino acids are increasingly recognized to be important in proteins that are involved in interactions with multiple different partners, for example in cell signalling and regulation of transcription (reviewed in (51)). Several functions have been postulated for these regions, including allowing highly specific interactions with fast on- and off-kinetics (52). KorA contains only a short, four amino acid, linker that allows extensive flexibility; giving similar entropic advantages to intrinsically disordered proteins. A highly flexible four amino acid linker has also been seen in the partitioning protein ParB from the plasmid P1, which allows the helix-turn helix domains to rotate essentially independently of the dimerization domains in different crystal structures (53). This allows the domains to contact several different arrangements of DNA binding sites, required for partitioning. Since proteins with such highly flexible linkers are hard to crystallize, they are likely to be under-reported. In addition servers predicting disorder use a large window size and hence will overlook short linkers. This study shows that even a four amino acid linker can be highly dynamic and indicates that flexibility and dynamics are as crucial as 3D structure in protein function.

## ACCESSION NUMBERS

The coordinates and diffraction data have been deposited with the Protein Data Bank: crystal structures of KorA 5CKT, KorA–O_A_ complex 1 5CM3, KorA–O_A_ complex 2 5CLV. The coordinates and NMR constraints for KorA have been deposited with the relevant databases with the PDB IDs: 2N5G and BMRB 6999.

## Supplementary Material

Supplementary Data

SUPPLEMENTARY DATA
