# Supplementary material for: Flexibility of KorA, a plasmid-encoded, global transcription regulator, in the presence and the absence of its operator
Source: Nucleic Acids Res. 2016 Mar 25;44(10):4947–56. doi: 10.1093/nar/gkw191 (PMC4889941; doi:10.1093/nar/gkw191)
Supplement: Supplementary Data [file gkw191_Supplementary_Data.zip › nar-02109-h-2015-File008.pdf]

Supplementary Information for

Flexibility of KorA, a plasmid-encoded, global transcription regulator, in the presence and the absence of its operator.

Karthik V. Rajasekar, Andrew Lovering, Felician Dancea, David J. Scott, Sarah Harris, Lewis E. H. Bingle, Manfred Roessle, Christopher M. Thomas, Eva I. Hyde and Scott A. White

Supplementary Figures 1 – 5

Supplementary Tables 1 – 2

Supplementary Movies 1 - 2

Supplementary References

A

```
TrbA  91 YERISVVLPSHKAFIVKKWGDDTRKKLRGRL 121
         : :...::: :.:.::.::  :..:: . .
KorA  71 YARVTAVLPEHQAYIVRKWEADAKKKQETKR 101
            .  .  ...  . :. .   . :.
 p53 326 EYFTLQIRGRERFEMFRELNEALELKDAQAG 356
```

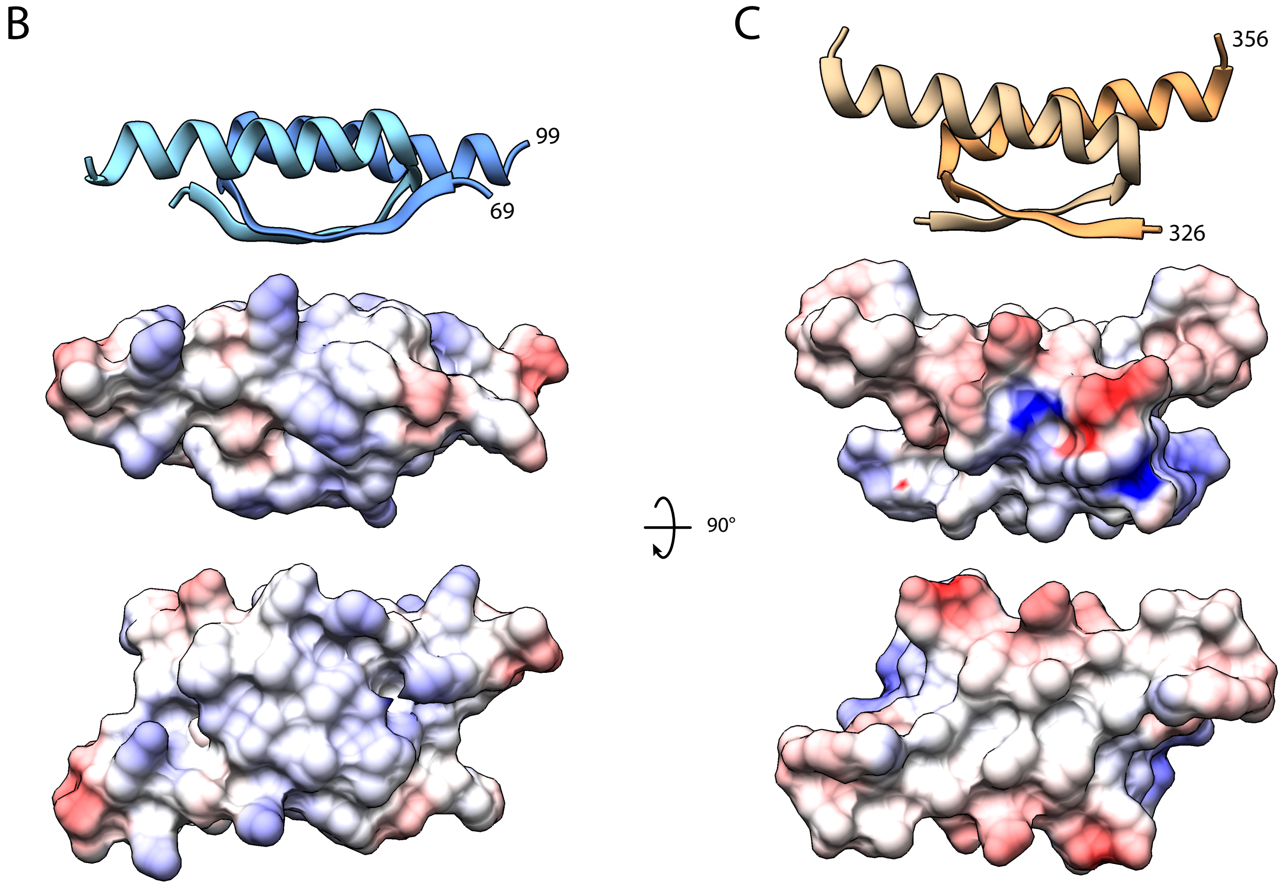

Supplementary Figure 1.

(**A**) Global sequence pair-wise alignments of the CTDs of TrbA with KorA (45.2% sequence identity, 77.4% sequence similarity) and KorA with p53 (6.5% sequence identity, 38.7% sequence similarity) using the program LALIGN (1).

Structural comparison of KorA CTD with that of p53.

(**B**) KorA (PDB:5CKT chains B and C).

(**C**) p53 (PDB:1AIE and symmetry mate x, 45.5-y, -z).
Top: ribbon diagram with residue numbers. Middle: electrostatic surface, same orientation (red negative, white neutral, blue positive). Bottom: rotated 90° with the p53 tetramerisation surface facing the viewer.

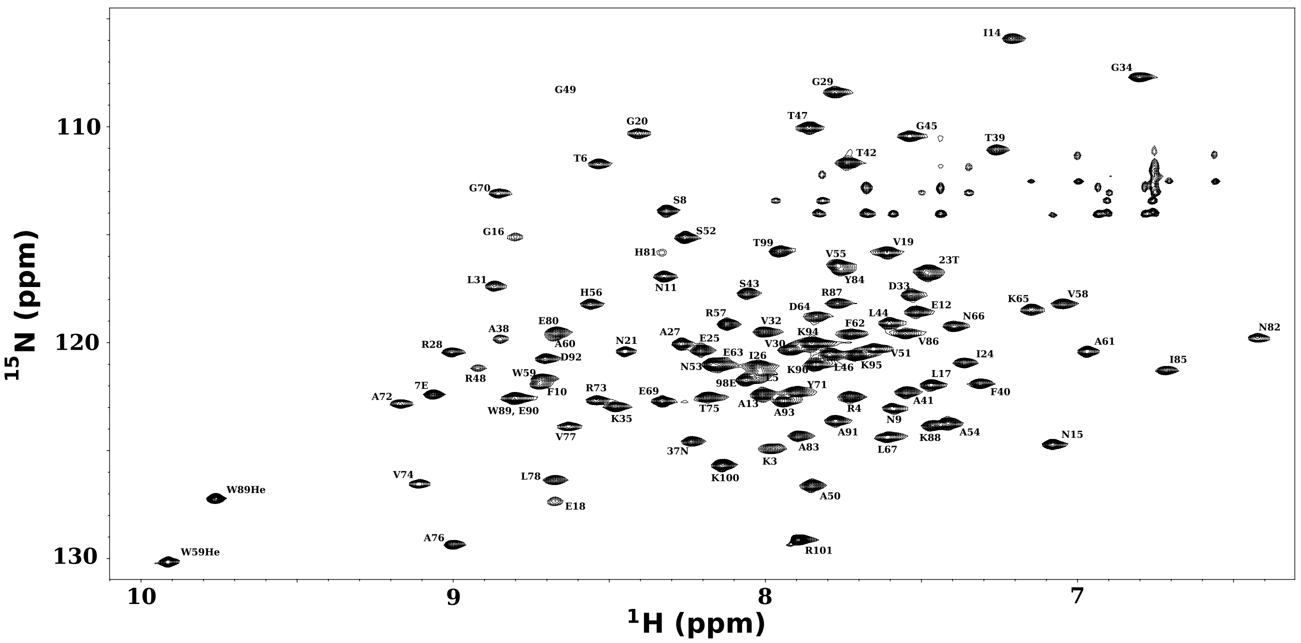

Supplementary Figure 2.

^{15}N-^{1}H HSQC of free KorA at 30 °C recorded at 600 MHz, in 20 mM sodium phosphate buffer pH 6.2, 100 mM NaCl and 1 mM EDTA. The peaks are labelled with the amino acid assignments of their NH group, the two peaks at approximately 10 ppm are from the indole NH groups of the two tryptophan residues.

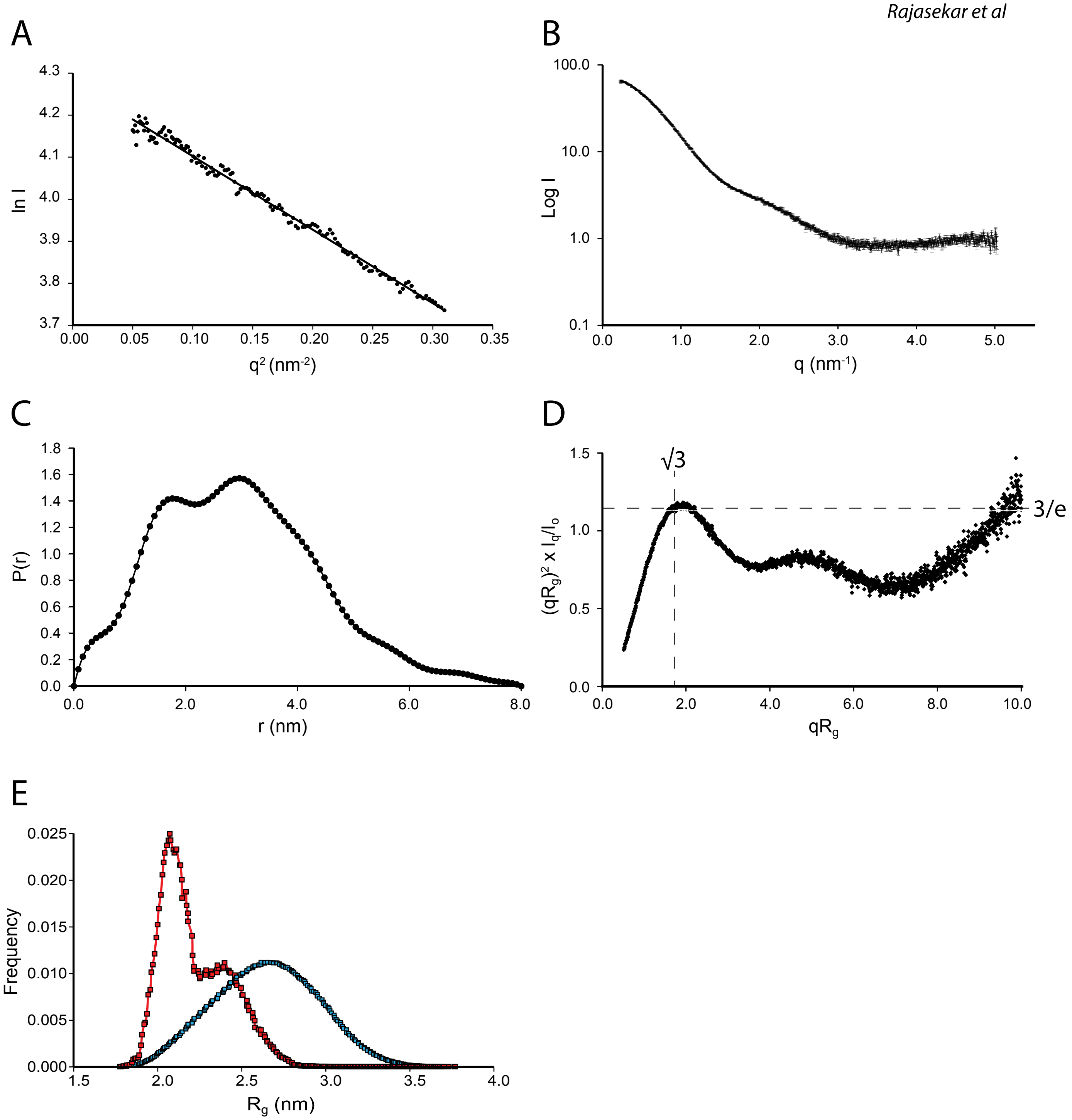

Supplementary Figure 3.

Small Angle X-ray Scattering data for free KorA in 20 mM Tris-HCl pH 7.0, 100 mM NaCl 1 mM EDTA buffer, at 20 °C.

(**A**) Guinier plot from scattering data in (B).

(**B**) Merged Small Angle Scattering curve for KorA at 2.5, 5.0 and 10 mg.ml^{-1}. The fit, over qR_g 0.54-1.30, gives $R_g = 2.30 \pm 0.006$ nm and $I_0 = 72.3 \pm 0.1$, leading to the molecular mass estimate of 21.3 kDa.

(**C**) Pairwise distribution plot (P(r) *vs* r) from the indirect Fourier transform of the scattering curve in B, calculated using GNOM (2). This has been fitted over the q range 0.227-4.89, and gives $R_g = 2.32$ nm and $I_0 = 72.3$ in good agreement with the Guinier plot.

(**D**) Dimensionless Kratky plot of $(qR_g)^2\ I_q/I_0$ *vs* qR_g for the scattering data in (B) (3)

(**E**) Plot of distribution of conformers *vs* R_g. Blue: distribution of R_gs for a pool of 10,000 random conformers. Red: distribution of 50 conformers that together best fit the SAXS curve, calculated by EOM 2.0 (4,5). The calculations were repeated 4 times, giving slightly different points, and the results smoothed.

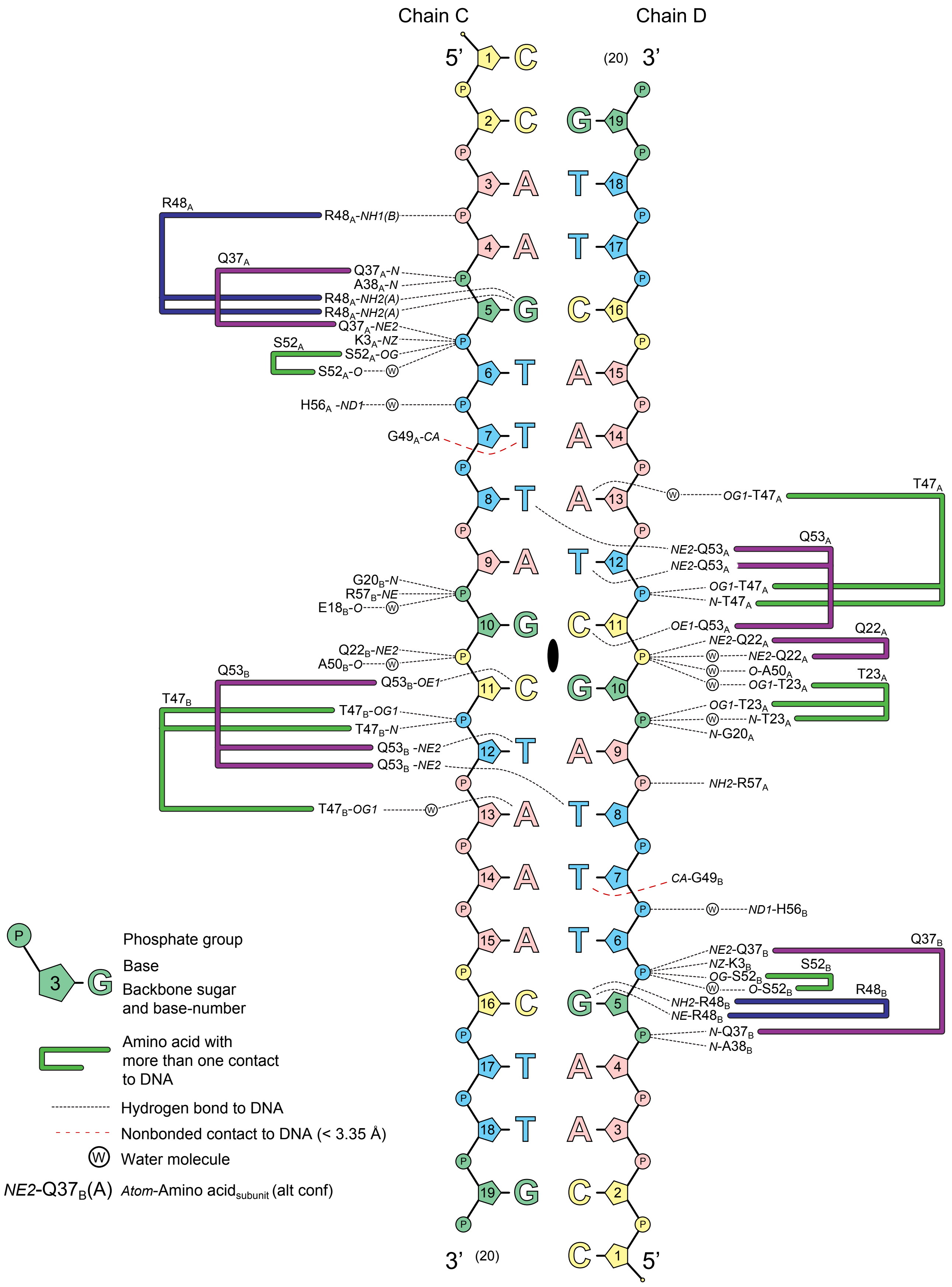

Supplementary Figure 4.

Schematic representation of the DNA-protein contacts for KorA–O_A complex, adapted from Nucplot (6). Nucleotides are numbered on the ribose ring, from 5' to 3' and shown in colour, A (pink), C (yellow), G (green) and T (blue). The base and ribose ring of G20 is not observed in any of the structures, while C1 makes a base triple with C2-G19 of a crystallographic symmetry mate. H-bonds to the DNA, whether direct or through water molecules (W), are shown by black dotted lines, with the amino acid, subunit and atom involved indicated. Red, dashed lines, show non-bonded contacts to the DNA. Amino acids showing more than one contact to the DNA are connected by coloured lines.

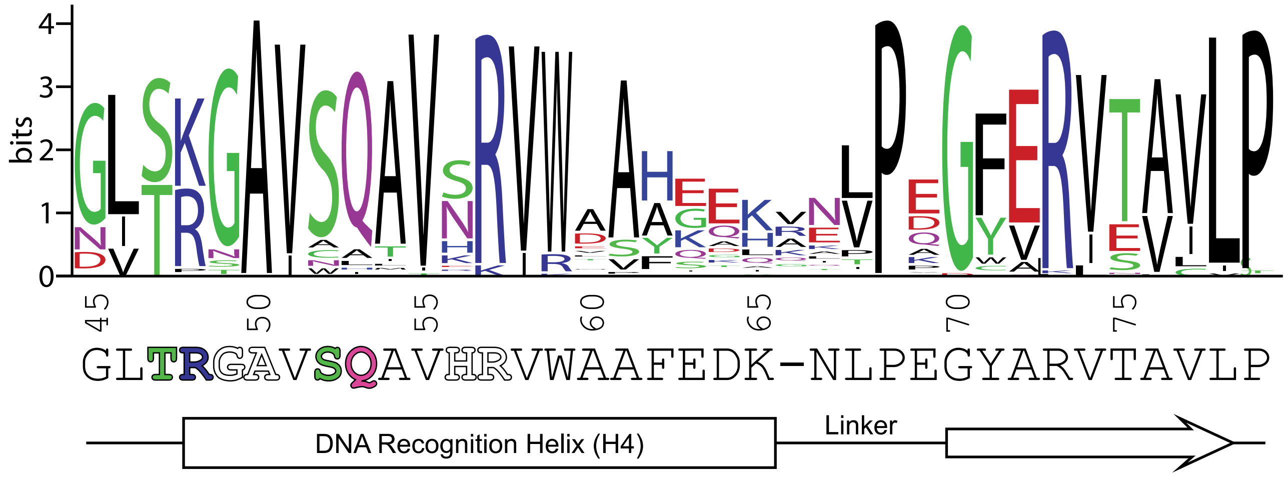

Supplementary Figure 5. Sequence conservation of residues 45-79 of KorB.

A sequence LOGO (7) of 92 KorA sequences with sequence identities greater than 37%, aligned by BLAST (8). The frequency of each amino acid at each position (as the $\log_2$ of its proportion/20) is shown by the height of each letter. Below: the sequence of KorA in RK9, and the secondary structure as a ribbon diagram. Aminoacids that contact the DNA are shown in outline, with those that make multiple contacts coloured as in Supplementary Figure 4.

Supplementary Table 1. Data collection and refinement statistics.

**PDB**	**PDB: 5CKT (apo form)**	**PDB: 5CM3 (KorA-O_A Cplx 1)**	**PDB: 5CLV (KorA-O_A Cplx 2)**
Beamline	ESRF ID14-2	ESRF BM30	ESRF BM30
Wavelength (Å)	0.933	0.9797	0.9797
Resolution range (Å) [a]	27.17 – 2.00 (2.07 - 2.00)	23.46 - 2.30 (2.38 - 2.30)	25.05 - 2.50 (2.59 - 2.50)
Space group	P 1	P 1 2_1 1	P 1 2_1 1
Unit cell (Å,°)	42.58 49.69 52.33 98.09 93.87 106.01	44.54 115.10 49.93 90 113.81 90	80.46 114.03 82.07 90 99.59 90
Total reflections [a]	105206 (10412)	38144 (3388)	93688 (9435)
Unique reflections [a]	26649 (2628)	19920 (1844)	49516 (5029)
Multiplicity [a]	3.9 (3.9)	1.9 (1.8)	1.9 (1.9)
Completeness (%)[a]	97.2 (96.3)	97.6 (90.1)	97.9 (99.8)
Mean I/sigma(I) [a]	26.1 (3.6)	17.4 (4.1)	12.4 (2.8)
Wilson B-factor ($Å^2$)	37.1	42.73	47.77
R-merge [a]	0.031 (0.438)	0.032 (0.277)	0.038 (0.352)
R-meas [a]	0.036 (0.507)	0.046 (0.391)	0.054 (0.498)
CC1/2 [a]	0.999 (0.821)	0.998 (0.545)	0.997 (0.834)
R-work [a]	0.193 (0.271)	0.193 (0.284)	0.280 (0.325)
R-free [a]	0.219 (0.293)	0.233 (0.313)	0.291 (0.755)
CC(work) [a]	0.956 (0.786)	0.944 (0.628)	0.888 (0.726)
CC(free) [a]	0.950 (0.808)	0.921 (0.678)	0.898 (1.000)
No. non-hydrogen atoms	3328	2429	7703
polypeptides	3112	1512	4427
DNA	-	778	3112
ligands	8	-	-
water	216	139	164
Protein residues	391	190	572
RMS bonds / angles	0.004 / 0.812	0.011 / 1.48	0.003 / 0.8
Ramachandran regions (%) favoured/allowed/outliers	98.2 / 1.3 / 0.5	98.4 / 1.1 / 0.5	96.1 / 3.0 / 0.9
Average B-factor ($Å^2$)	55.86	60.55	38.50
polypeptide	56.43	60.79	41.06
DNA		61.16	35.36
ligands	41.84	-	-
solvent	48.25	54.43	28.43

[a] Statistics for the highest-resolution shell are shown in parentheses.

Supplementary Table 2 NMR constraints and refinement statistics

**NMR distance and dihedral constraints**	**PDB: 2N5G**
**Distance constraints**	
Total NOE	2588
Inter monomer	38
Ambiguous	124
Intra-residue	1027
Inter-residue	1523
Sequential ($\|i – j\| = 1$)	850
Medium-range ($\|i – j\| < 4$)	476
Long-range ($\|i – j\| > 5$)	197
Hydrogen bonds	4
Total dihedral angle restraints	304
ϕ / ψ	152 / 152
**Structure statistics**	
Violations (mean and s.d.)	
Distance constraints (Å)	0.021 $\pm$ 0.003
Dihedral angle constraints (º)	0.40 $\pm$ 0.06
Max. dihedral angle violation (º)	1.06
Max. distance constraint violation (Å)	0.352
Deviations from idealized geometry	
Bond lengths (Å)	0.0033 $\pm$ 0.0001
Bond angles (º)	0.45 $\pm$ 0.02
Impropers (º)	1.21 $\pm$ 0.08
Ramachandran regions (%) favoured/allowed/outliers	93.8 / 5.8 / 0.4
Average pairwise r.m.s. deviation** (Å)	
Backbone	
Monomer 1, AA 6-66	0.54 $\pm$ 0.11
Monomer 2, AA 6-66	0.58 $\pm$ 0.11
Monomer 1 & 2, AA 72-98	1.04 $\pm$ 0.33

Supplementary Movie 1.

MD simulations of KorA-O_A Complex 1 sampled over 100 ns. The KorA-O_A complex is viewed in the same orientation, with the same colours, as Figure 3B.

Supplementary Movie 2.

MD simulations of the collapse of the free KorA dimer, sampled over 100 ns. The initial KorA structure is that of the protein only PDB:5CM3, but in the absence of DNA. The movie is viewed in the same orientation and the same colours as Figure 3A.

Supplementary References

1. Huang, X. and Miller, W. (1991) A time-efficient, linear-space local similarity algorithm. *Advances in Applied Mathematics*, **12**, 337-357.
2. Semenyuk, A.V. and Svergun, D.I. (1991) GNOM - a program package for small-angle scattering data processing. *Journal of Applied Crystallography*, **24**, 537-540.
3. Durand, D., Vives, C., Cannella, D., Perez, J., Pebay-Peyroula, E., Vachette, P. and Fieschi, F. (2010) NADPH oxidase activator p67(phox) behaves in solution as a multidomain protein with semi-flexible linkers. *Journal of structural biology*, **169**, 45-53.
4. Bernado, P., Mylonas, E., Petoukhov, M.V., Blackledge, M. and Svergun, D.I. (2007) Structural characterization of flexible proteins using small-angle X-ray scattering. *J Am Chem Soc*, **129**, 5656-5664.
5. Petoukhov, M.V., Franke, D., Shkumatov, A.V., Tria, G., Kikhney, A.G., Gajda, M., Gorba, C., Mertens, H.D., Konarev, P.V. and Svergun, D.I. (2012) New developments in the program package for small-angle scattering data analysis. *J Appl Crystallogr*, **45**, 342-350.
6. Luscombe, N.M., Laskowski, R.A. and Thornton, J.M. (1997) NUCPLOT: a program to generate schematic diagrams of protein-nucleic acid interactions. *Nucleic Acids Res*, **25**, 4940-4945.
7. Crooks, G.E., Hon, G., Chandonia, J.M. and Brenner, S.E. (2004) WebLogo: a sequence logo generator. *Genome research*, **14**, 1188-1190.
8. Altschul, S.F., Madden, T.L., Schäffer, A.A., Zhang, J., Zhang, Z., Miller, W. and Lipman, D.J. (1997) Gapped BLAST and PSI-BLAST: a new generation of protein database search programs. *Nucleic Acids Research*, **25**, 3389-3402.
