# Supplementary material for: Flexibility of KorA, a plasmid-encoded, global transcription regulator, in the presence and the absence of its operator
Source: Nucleic Acids Res. 2016 Mar 25;44(10):4947–56. doi: 10.1093/nar/gkw191 (PMC4889941; doi:10.1093/nar/gkw191)
Supplement: SUPPLEMENTARY DATA [file supp_44_10_4947__index.html]

Flexibility of KorA, a plasmid-encoded, global transcription regulator, in the presence and the absence of its operator — Flexibility of KorA, a plasmid-encoded, global transcription regulator, in the presence and the absence of its operator — SUPPLEMENTARY DATA 

# Flexibility of KorA, a plasmid-encoded, global transcription regulator, in the presence and the absence of its operator

## SUPPLEMENTARY DATA

- SUPPLEMENTARY DATA
- SUPPLEMENTARY DATA
- SUPPLEMENTARY DATA
